# Bilateral Encephalitozoon hellem Keratoconjunctivitis With Microsporidial Spores in Parrot Feces

**DOI:** 10.7759/cureus.113157

**Published:** 2026-07-22

**Authors:** Xu Wang, Chao Wang, Jing Gao, Li Li

**Affiliations:** 1 School of Clinical Medicine, Jining Medical University, Jining, CHN; 2 Department of Ophthalmology, Zaozhuang Municipal Hospital, Zaozhuang, CHN

**Keywords:** avian exposure, corneal scraping, encephalitozoon hellem, giemsa staining, immunocompetent patient, metagenomic next-generation sequencing, microsporidial keratoconjunctivitis, pet parrot

## Abstract

We describe bilateral *Encephalitozoon hellem* keratoconjunctivitis in a 39-year-old immunocompetent woman with two years of daily close contact with a pet parrot. She presented with a three-week history of bilateral ocular redness, itching, foreign-body sensation, and blurred vision that had not improved with topical fluorometholone, lubricants, and levofloxacin prescribed for presumed dry eye disease. Slit-lamp examination showed bilateral conjunctival inflammation and diffuse superficial punctate corneal infiltrates. Giemsa staining of a left-eye corneal scraping demonstrated oval spore-like structures, while bacterial and fungal cultures were negative. Metagenomic next-generation sequencing (mNGS) of the same specimen detected 9,684 reads assigned to *E. hellem*, with a relative abundance of approximately 98% and genome coverage of 63%, supporting the diagnosis. Fluorescence microscopy of a fecal specimen from the pet parrot revealed a small number of spore-like structures morphologically compatible with microsporidia, but no molecular typing was performed. The symptoms and corneal lesions resolved over six weeks during treatment with topical 0.02% polyhexamethylene biguanide, topical 0.5% gatifloxacin administered as post-scraping antibacterial prophylaxis, and a short course of oral albendazole, with no recurrence during four months of follow-up. This case highlights the diagnostic value of combining corneal-scraping microscopy with mNGS in treatment-refractory keratoconjunctivitis and the importance of obtaining a detailed avian-exposure history. It also illustrates that microscopic findings in avian feces alone cannot establish zoonotic transmission.

## Introduction

Microsporidia are obligate intracellular eukaryotic parasites that cause ocular disease primarily in the form of keratoconjunctivitis or stromal keratitis [1]. Microsporidial keratoconjunctivitis (MKC) is increasingly recognized in immunocompetent individuals. It usually presents with non-purulent conjunctivitis and multifocal punctate epithelial keratitis and may be mistaken for viral or immune-mediated disease. Because microsporidia cannot be recovered on routine bacterial or fungal culture media, diagnosis may be delayed. Reported non-avian risk factors include contaminated water or soil, ocular trauma, and contact-lens wear [2].

Recent reports associate parrot exposure with *Encephalitozoon hellem* MKC, but few studies have included species-level testing of avian specimens [3-5]. Corneal-scraping microscopy can detect spores but not identify species, whereas metagenomic next-generation sequencing (mNGS) can identify difficult-to-culture organisms at the species level without a prespecified target [2,4]. We report bilateral MKC in an immunocompetent woman with prolonged daily contact with a pet parrot. Giemsa staining of a left-eye corneal scraping showed spore-like structures, and mNGS identified *E. hellem*. Fluorescence microscopy of parrot feces showed spore-like structures morphologically compatible with microsporidia, but molecular typing was not performed. Microscopy and molecular speciation were complementary in this case, but the untyped avian finding does not establish zoonotic transmission.

## Case presentation

Patient information

A 39-year-old woman who worked as an office clerk presented with a three-week history of bilateral ocular redness, itching, foreign-body sensation, and blurred vision. She was otherwise healthy, denied any systemic complaints, and reported no history of immunodeficiency, surgery, or trauma. She had kept a pet parrot at home for two years and had daily close contact with it. Her husband and son lived in the same household but did not have frequent direct contact with the bird. Both were examined in our clinic. Neither reported ocular redness, foreign-body sensation, or other ocular discomfort, and slit-lamp examinations showed no conjunctival congestion or corneal epithelial lesions. Before referral, the patient had been diagnosed with dry eye disease at another hospital and had received fluorometholone, sodium hyaluronate, and levofloxacin eye drops without clinical improvement.

Ocular examination

**Figure 1:**
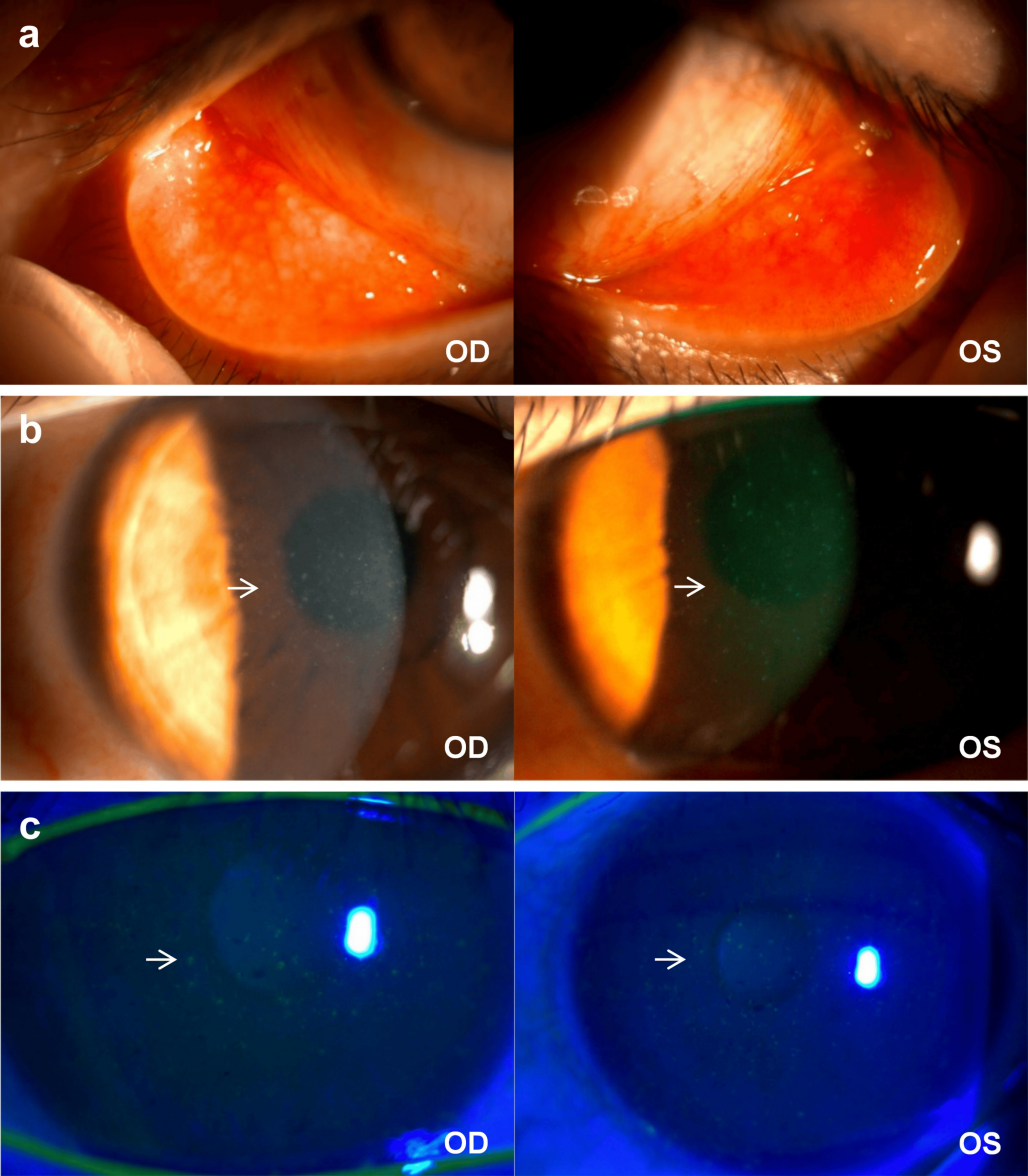
Ocular signs at initial presentation (a) Slit-lamp photographs of the everted lower eyelids show bilateral mixed follicular-papillary conjunctival reactions. (b) Anterior-segment slit-lamp photographs show diffuse superficial punctate corneal epithelial infiltrates. (c) Cobalt-blue illumination after fluorescein instillation shows diffuse punctate epithelial staining in both corneas. Arrows indicate representative infiltrates in (b) and representative areas of epithelial staining in (c). Original magnification: ×16 in all panels OD, right eye; OS, left eye.

Uncorrected visual acuity (UCVA), measured using a standard logarithmic visual acuity chart at 5 m, was 0.8 decimal (0.10 logMAR) in both eyes. Pinhole testing did not improve visual acuity. Intraocular pressure, measured using a non-contact air-puff tonometer, was within the normal range bilaterally. Anterior segment examination showed bilateral mixed conjunctival and ciliary injection, with papillae and follicles on the lower palpebral conjunctiva (Figure 1a). There was no ocular discharge, membrane or pseudomembrane formation, and chemosis or preauricular lymphadenopathy. Corneal examination showed bilateral superficial punctate infiltrates, mainly in the central cornea (Figure 1b). Fluorescein staining revealed diffuse punctate epithelial staining in both eyes (Figure 1c). The anterior chamber, iris, and lens were unremarkable.

Diagnostic assessment

The initial working diagnosis was keratoconjunctivitis of undetermined cause.

Adenoviral keratoconjunctivitis, particularly epidemic keratoconjunctivitis (EKC), was first considered because the patient had bilateral conjunctival inflammation and corneal epithelial lesions. However, she had no known contact with patients with keratoconjunctivitis. She also had no watery discharge, subconjunctival hemorrhage, membrane or pseudomembrane formation, or preauricular lymphadenopathy. The corneal infiltrates were already present at the initial examination, rather than appearing after resolution of epithelial disease, which was less consistent with EKC.

Thygeson's superficial punctate keratitis (TSPK) was also considered because of the bilateral superficial punctate corneal lesions. However, the prominent conjunctival congestion, papillae, and follicles were atypical for TSPK. The disease had not followed a chronic relapsing course, and the patient had not improved after topical fluorometholone. These findings made TSPK less likely.

Dry eye disease was less likely because the prominent follicular-papillary conjunctival reaction was atypical, and the symptoms had not improved with sodium hyaluronate. Drug-induced toxic keratopathy could not explain the onset because the symptoms preceded the prescribed topical medications. In treatment-refractory keratoconjunctivitis, the combination of pet-parrot exposure and superficial punctate epithelial infiltrates should raise suspicion for MKC and prompt corneal scraping for microbiological examination [2-5].

**Figure 2:**
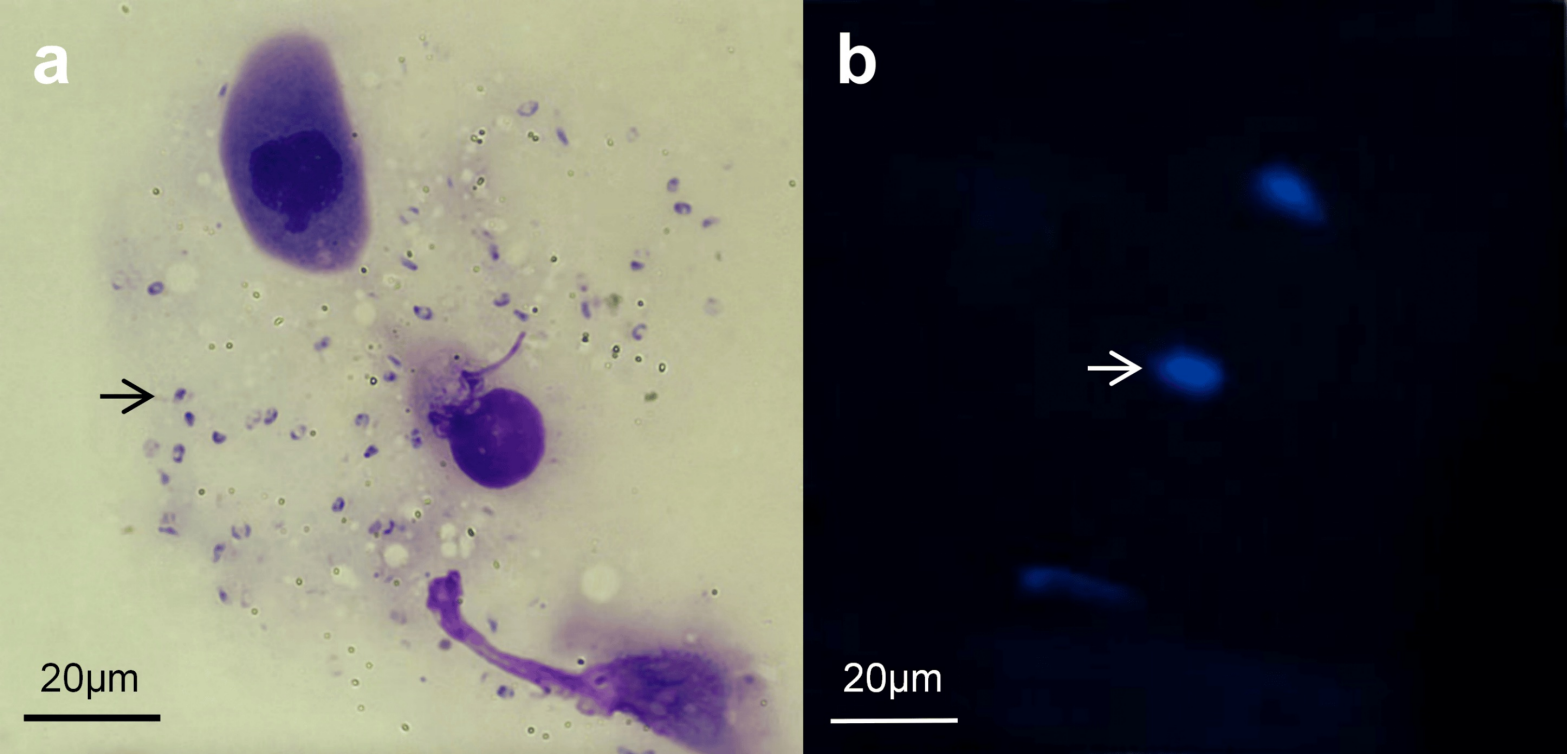
Microscopic findings from the corneal scraping and pet parrot fecal specimen (a) Giemsa-stained corneal scraping from the left eye, showing bluish-purple oval spore-like structures adjacent to epithelial cells (black arrow). (b) Fluorescence microscopy of a fecal specimen from the patient’s pet parrot, showing a small number of blue fluorescent spore-like structures morphologically compatible with microsporidia; the white arrow indicates a representative structure. Original magnification: ×1000 for both panels; scale bars: 20 μm

Accordingly, a corneal scraping was obtained from the left eye for microbiological assessment. Only one eye was sampled to avoid bilateral epithelial injury. Giemsa staining showed scattered bluish-purple, oval, spore-like structures adjacent to epithelial cells (Figure 2a). Bacterial and fungal cultures were negative.

The corneal scraping sample was then submitted for mNGS. The assay met the laboratory’s quality criterion: 95.35% of the sequencing data had a base-call accuracy of at least 99.9%, above the acceptance threshold of 85%. A total of 9,684 species-level reads were assigned to *E. hellem*, with genome coverage of 63.5%. After host-read removal, *E. hellem* accounted for 98.4% of species-level reads within the corresponding microbial category. The detected bacterial taxa had low species-level read counts and genome coverage ranging from 0.23% to 0.91% and were classified by the laboratory as suspected background microorganisms. No other clinically relevant pathogen was detected.

The report did not provide a separate organism-specific positivity cutoff. The clinical relevance of *E. hellem* was therefore determined by its concordance with the spore-like structures observed on Giemsa staining and the patient’s clinical presentation, rather than by the mNGS result alone. Together, the microscopy and mNGS findings supported the diagnosis of *E. hellem* MKC in the sampled left eye.

Fluorescence microscopy of a fecal specimen from the patient’s pet parrot revealed a small number of blue fluorescent spore-like structures morphologically compatible with microsporidia (Figure 2b); no species-level molecular testing was performed on the avian specimen.

Timeline

The patient's clinical course, including key findings, treatment milestones, and follow-up outcomes, is summarized chronologically in Table 1.

**Table 1:** Clinical timeline PHMB, polyhexamethylene biguanide; UCVA, uncorrected visual acuity.

Time point	Findings and management
Initial presentation	Bilateral conjunctival injection with mixed follicular-papillary reaction and diffuse superficial punctate epithelial infiltrates; diffuse punctate fluorescein staining in both eyes. UCVA was 0.8 decimal (0.10 logMAR) in both eyes.
Treatment initiation	Giemsa staining of a left-eye corneal scraping showed spore-like structures, and metagenomic next-generation sequencing detected *Encephalitozoon hellem*. Topical 0.02% PHMB and 0.5% gatifloxacin were administered to both eyes six times daily, and oral albendazole 400 mg twice daily was initiated.
Treatment day 10	Punctate staining decreased and was confined mainly to the inferior central corneas. Albendazole was discontinued; PHMB and gatifloxacin were reduced to four times daily.
Treatment week 3	The corneal lesions had nearly resolved, with only scattered peripheral punctate staining remaining. UCVA was 1.0 decimal (0.00 logMAR) in both eyes. PHMB and gatifloxacin were continued.
Treatment week 6	Both corneas were clear, with no fluorescein staining. UCVA was 1.2 decimal (−0.08 logMAR) in both eyes. All medications were discontinued.
Month 4	The patient remained asymptomatic, with no recurrence. No further treatment was required.

Treatment course

After microsporidial infection was confirmed four days after presentation, the patient began combination therapy. The initial regimen included topical 0.02% polyhexamethylene biguanide (PHMB), one drop in both eyes six times daily, and topical 0.5% gatifloxacin, one drop in both eyes six times daily. Gatifloxacin was used as empirical antibacterial prophylaxis after corneal scraping. Oral albendazole was administered at 400 mg twice daily.

On treatment day 10, after symptomatic improvement, oral albendazole was discontinued. Topical PHMB and gatifloxacin were reduced to one drop in both eyes four times daily. Topical treatment was continued for a total of six weeks.

Follow-up and outcomes

**Figure 3:**
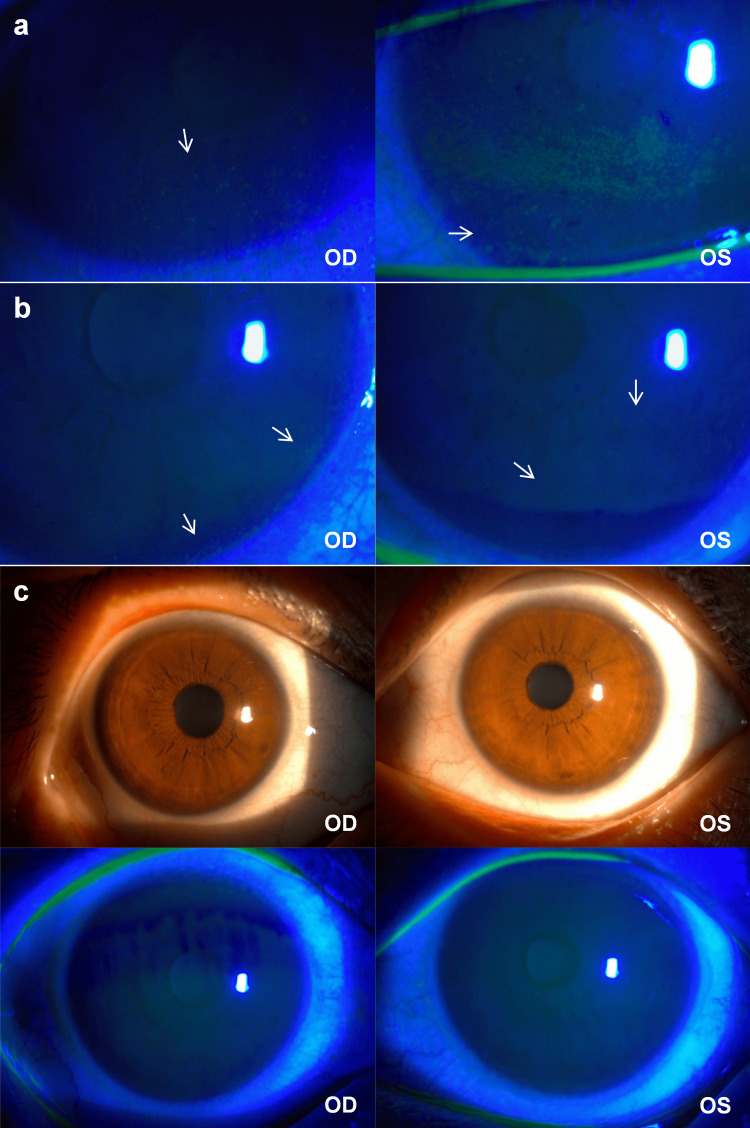
Serial ocular findings during treatment and follow-up (a) On treatment day 10, cobalt-blue illumination after fluorescein instillation shows decreased punctate epithelial staining, largely confined to the inferior central cornea. The more confluent staining in the left eye corresponds to the corneal-scraping site. (b) At week 3, only a few scattered peripheral fluorescein-positive epithelial lesions remain. Arrows in (a) and (b) indicate representative areas of residual epithelial staining. (c) At week 6, slit-lamp photographs (upper row) show clear corneas, and cobalt-blue photographs after fluorescein instillation (lower row) show negative staining in both eyes. Original magnification: ×16 in all panels OD, right eye; OS, left eye.

On treatment day 10, the patient reported improvement in ocular symptoms. Slit-lamp examination showed decreased diffuse punctate corneal epithelial staining, which was confined to the inferior central cornea (Figure 3a).

At treatment week 3, the corneal infiltrates had nearly resolved. Fluorescein staining showed only a few scattered punctate lesions in the peripheral cornea (Figure 3b). UCVA improved to 1.0 decimal (0.00 logMAR) in both eyes.

At treatment week 6, both corneas were clear, and fluorescein staining was negative (Figure 3c). UCVA improved further to 1.2 decimal (-0.08 logMAR) bilaterally, compared with 0.8 decimal (0.10 logMAR) at presentation. Because the symptoms had resolved and the ocular findings had normalized, all medications were discontinued.

The patient tolerated treatment well and reported no local or systemic adverse reactions. After diagnosis, the pet parrot was removed from the household, and the patient had no further direct contact with it. During four months of follow-up, she remained asymptomatic, with no recurrence. No similar ocular symptoms were reported by other household members.

## Discussion

Bilateral MKC has previously been reported in immunocompetent patients [6,7]. In this case, mNGS identified *E. hellem* in a corneal specimen from the left eye. Fluorescence microscopy of the patient’s parrot feces showed only spore-like structures morphologically compatible with microsporidia. The human and avian findings therefore provide different levels of evidence and should be interpreted separately.

Avian exposure and interpretation of the fecal finding

Exposure has been reported in *E. hellem* ocular infection and extraintestinal *E. cuniculi* infection in bird owners [8,9]. These reports support an association between avian contact and human microsporidiosis, but they do not establish a single transmission route. More recent reports have described *E. hellem* keratoconjunctivitis in parrot owners. In the cases reported by Zhang et al. and Liang and Zhang, parrot exposure was documented, but species-level testing of avian specimens was unavailable [3,5]. Sun et al., however, identified *E. hellem* by mNGS in affected patients and epidemiologically linked parrots [4]. Their findings provide stronger molecular support for a possible avian source.

The present case falls between these levels of evidence. The parrot was examined, but the fecal finding was based on microscopy alone. This result adds epidemiological context to the patient’s exposure history, but it cannot identify the source of infection. Molecular species determination and comparison of human and avian sequences would be needed to provide stronger evidence of a shared pathogen.

The fecal finding could represent active avian infection, asymptomatic carriage, transient passage of environmental spores, specimen contamination, or exposure of both hosts to a shared environmental source. Household contacts had no ocular symptoms, and the patient had no recurrence after the parrot was removed from the household and direct contact ceased. These observations are reassuring, but they do not establish the direction of transmission or show that cessation of exposure prevented recurrence. Pet-bird exposure should therefore be documented during history-taking, and targeted animal or environmental assessment may be considered when indicated. Morphological evidence alone is insufficient to attribute zoonotic transmission.

Diagnostic implications

The investigations were most informative when interpreted in sequence. The initial bilateral conjunctival inflammation and diffuse punctate epithelial keratitis were non-specific and overlapped with viral, toxic, and immune-mediated ocular surface disease [2]. Examination of a Giemsa-stained corneal scraping provided the first direct microbiological evidence by showing spore-like structures [2,10]. Microscopy alone, however, could not determine the causative species. mNGS subsequently provided species-level identification of *E. hellem* in the sampled corneal specimen. The use of mNGS as a complementary diagnostic method in infectious keratitis has been described previously [11].

Because corneal scraping was performed only on the left eye, species-level confirmation is limited to that eye. Involvement of the right eye was inferred from the closely matched clinical findings and synchronous response to the same treatment regimen. In this case, mNGS followed direct microscopic evidence and was used for confirmation and species identification rather than as a stand-alone screening test. This sequence may be useful when routine cultures are negative, corneal material is limited, or the clinical appearance is insufficiently specific.

Treatment and clinical outcome

Treatment of superficial MKC remains empirical, and spontaneous resolution has been reported in immunocompetent patients [2,12]. After microbiological confirmation, the patient received topical PHMB, oral albendazole, and prophylactic topical gatifloxacin. The corneal epithelial lesions resolved gradually while the patient was receiving this regimen. Because the treatments overlapped and superficial disease may be self-limiting, recovery cannot be attributed to any single agent.

The contribution of topical PHMB remains uncertain. A randomized placebo-controlled trial found no significant difference in time to clinical resolution between PHMB and placebo for MKC [13]. Evidence supporting systemic albendazole for isolated superficial ocular infection is also limited [2,12]. Albendazole was discontinued after 10 days, while clinical improvement continued during topical therapy. This clinical course does not permit a firm conclusion regarding the effect of albendazole. Topical gatifloxacin was intended to reduce the risk of secondary bacterial infection and was not used as specific anti-microsporidial treatment.

The favorable outcome in this patient should not be generalized to microsporidial stromal keratitis. Stromal disease is a distinct and more persistent clinical entity that may require prolonged medical therapy or surgical intervention [14]. The therapeutic observations in this report therefore apply specifically to superficial epithelial keratoconjunctivitis in an immunocompetent patient.

Limitations

This report has three main limitations. First, the parrot fecal specimen was examined only by microscopy. Molecular identification, quantification, and sequence comparison with the human ocular specimen were unavailable, and no cage, water, or other environmental samples were examined. Second, the human diagnosis was based on microscopy and mNGS without confirmation by targeted PCR or Sanger sequencing. In addition, only the left eye underwent corneal scraping. Third, combination therapy and the four-month follow-up period limit conclusions regarding treatment efficacy and long-term recurrence. Together, the microscopy and mNGS findings support the diagnosis in the sampled eye. The epidemiological and therapeutic findings should nevertheless be interpreted within these limitations.

## Conclusions

We report bilateral epithelial MKC in an immunocompetent adult. mNGS identified *E. hellem* in a corneal scraping from the left eye after Giemsa staining showed spore-like structures. Involvement of the fellow eye was inferred clinically from the matched phenotype and synchronous response in both eyes. A small number of spore-like structures morphologically compatible with microsporidia were observed in feces from the patient’s pet parrot. Because the avian specimen was not molecularly typed, species matching and source attribution were not possible. The ocular lesions resolved over six weeks during combination therapy. However, superficial disease may be self-limiting, and several agents were administered concurrently. The outcome therefore cannot be attributed to a specific treatment. In patients with persistent keratoconjunctivitis and a history of avian exposure, microscopy of corneal scrapings followed by molecular speciation may aid diagnosis. Microscopy of animal specimens alone cannot establish zoonotic transmission.
